# Short- and Long-Term Neurodevelopmental Outcomes of Very Preterm Infants with Neonatal Sepsis: A Systematic Review and Meta-Analysis

**DOI:** 10.3390/children6120131

**Published:** 2019-12-01

**Authors:** Shirley Cai, Deanne K. Thompson, Peter J. Anderson, Joseph Yuan-Mou Yang

**Affiliations:** 1 Victorian Infant Brain Study, Murdoch Children’s Research Institute, Royal Children’s Hospital, Flemington Road, Parkville, VIC 3052, Australia; shirleycai6894@gmail.com (S.C.); peter.j.anderson@monash.edu (P.J.A.); 2 Developmental Imaging, Murdoch Children’s Research Institute, Parkville, VIC 3052, Australia; Joseph.Yang4@rch.org.au; 3 Melbourne Medicine School, Faculty of Medicine, Dentistry and Health Sciences, The University of Melbourne, Parkville, VIC 3052, Australia; 4 Department of Paediatrics, The University of Melbourne, Parkville, VIC 3052, Australia; 5 Florey Institute of Neuroscience and Mental Health, Parkville, VIC 3052, Australia; 6 Turner Institute for Brain and Mental Health, School of Psychological Sciences, Monash University, Clayton, VIC 3800, Australia; 7 Neuroscience Research, Murdoch Children’s Research Institute, Parkville, VIC 3052, Australia; 8 Department of Neurosurgery, Royal Children’s Hospital, Parkville, VIC 3052, Australia

**Keywords:** premature, brain, infection, infant, development, cognition

## Abstract

Sepsis is commonly experienced by infants born very preterm (<32 weeks gestational age and/or <1500 g birthweight), but the long-term functional outcomes are unclear. The objective of this systematic review was to identify observational studies comparing neurodevelopmental outcomes in very preterm infants who had blood culture-proven neonatal sepsis with those without sepsis. Twenty-four studies were identified, of which 19 used prespecified definitions of neurodevelopmental impairment and five reported neurodevelopmental outcomes as continuous variables. Meta-analysis was conducted using 14 studies with defined neurodevelopmental impairment and demonstrated that very preterm infants with neonatal sepsis were at higher risk of impairments, such as cerebral palsy and neurosensory deficits, compared with infants without sepsis (OR 3.18; 95% CI 2.29–4.41). Substantial heterogeneity existed across the studies (*I*^2^ = 83.1, 95% CI 73–89). The five studies that reported outcomes as continuous variables showed no significant difference in cognitive performance between sepsis and non-sepsis groups. Neonatal sepsis in very preterm infants is associated with increased risk of neurodevelopmental disability. Due to the paucity of longitudinal follow-up data beyond 36 months, the long-term cognitive effect of neonatal sepsis in very preterm infants could not be conclusively determined. Effects on the development of minor impairment could not be assessed, due to the small numbers of infants included in the studies.

## 1. Introduction

Sepsis is a clinical condition characterised by bacteraemia and clinical signs of systemic infection [1]. It is one of the events that can occur during neonatal intensive care unit (NICU) admission and contributes significantly to the morbidity of very preterm (VP: <32 weeks gestational age) and/or very low birthweight (VLBW: <1500 g) infants [2,3,4]. Studies have reported rates of sepsis are inversely proportional to gestational age, with 33% of infants born less than 28 weeks acquiring sepsis compared with 60% of infants born less than 25 weeks [5].

During the neonatal period, complications such as sepsis can have dramatic effects on the growth and development of the child, especially in children born very prematurely [6,7]. The mechanism of how sepsis inflicts brain damage has been hypothesised. Research suggests the developing brain is vulnerable to the systematic inflammatory milieu characteristic of sepsis, as well as cytotoxic and ischaemic injury from hypotension and reduced cerebral blood flow [7]. Together, these factors may result in white matter abnormalities and diffuse injury to premyelinating oligodendrocytes, which have been shown to be closely associated with increased risk for impaired cognitive and motor functioning [8,9]. Such morbidities are complex and can range from major impairments, such as cerebral palsy, to more subtle deficits such as difficulties with memory and attention. Regardless, they all have the potential to affect a child’s academic, social and emotional functioning. Neurodevelopmental follow-up in VP infants with sepsis is crucial for the early identification of developmental delay so that targeted interventions can be prescribed to minimise long-lasting impairments. 

The short-term cognitive impact of sepsis in VP infants has been studied. A systematic review of 17 studies on VLBW infants with neonatal sepsis concluded these infants are at twice the risk of neurodevelopmental impairment compared with their non-sepsis counterparts [10]. The follow-up duration of studies included in this review ranged from 6 to 60 months; 11 of the 17 studies had a follow-up duration of less than 36 months. Many studies focused on serious disabilities, such as cerebral palsy and neurosensory impairment, using dichotomised definitions of neurodevelopmental impairment (NDI) that were study-specific. Little is known about NDI beyond 36 months and whether they impact other cognitive domains.

A comprehensive analysis of the long-term consequences of sepsis in children born VP is needed to better inform parents and health professionals of the long-term cognitive risks and to guide targeted and age-specific interventions in high-risk infants to achieve optimal function.

The objective of this systematic review was to provide an updated review of all VP cohort studies, which evaluated long-term neurodevelopmental outcomes beyond 18 months of age in infants who had culture-positive neonatal sepsis compared with VP infants without sepsis.

## 2. Materials and Methods

The review protocol consisted of a detailed research question, search strategy, initial screening criteria and full-text screening criteria. Using the PICOS (population, intervention, comparator, outcome, study design) approach, the research question was formulated as follows: “*Does postnatal sepsis in VP infants adversely impact long-term neurodevelopmental outcomes compared with VP infants without sepsis exposure?*”.

### 2.1. Literature Search Strategy

An electronic search strategy was used to identify relevant studies in the following databases by one reviewer (S.C.): Medline (using Ovid, 1966–present), Embase (using Ovid, 1980–present) and PubMed (1966–present). The search strategy for the Medline database comprised the following Medical Subject Headings (MeSH) or keywords, using Boolean terms ‘or’ then combined with ‘and’: (1) infant, premature *or* infant, low birthweight; (2) and sepsis; (3) and neurodevelopmental disorders *or* developmental disabilities or intellectual disability *or* learning disorders or motor skills disorders. The search was then adapted for each database and the last search was conducted on 24 February 2018, see Table S1 for the full search strategy. Additional article screening was performed by hand search of the reference lists of selected articles.

### 2.2. Study Selection Criteria

After removing duplicated studies, the search results were screened using titles and abstracts. Full texts of the selected articles were then reviewed independently by one reviewer (S.C.). The articles were included if they were full-text academic journal articles that reported quantitative information on our PICOS parameters in observational clinical studies.

Studies had to meet the following selection criteria for inclusion: (1) Participants comprised of infants born <32 weeks’ gestational age and/or <1500 g birth weight with no major genetic or congenital abnormalities; (2) blood culture-proven sepsis during the NICU admission, with optional additional criteria for sepsis definition as per study; and (3) follow-up duration of at least 18 months assessing neurodevelopment. We intended to include studies with a longer follow-up duration, starting at 18 months, and had no restriction on the upper limit of follow-up duration.

Additional study inclusion criteria included: (1) Studies reporting original data; (2) if there were studies that used the same outcome data from the same institution, the one with greater information was included; (3) studies that included VP infants with sepsis as part of a larger preterm cohort and contained sufficient outcome information; (4) studies published in English or had been translated into English. Study exclusion criteria included: (1) Review articles, nonanalytical studies and expert opinion articles; (2) studies where outcomes were not reported; (3) studies where outcomes of the sepsis exposure group could not be separated from those of the non-sepsis exposure group. No restriction on publication or publication status was applied.

### 2.3. Data Extraction

The information extracted from studies included: study design (single vs multi-centre; case-control vs. case-cohort studies), year of birth, number of infants with and without sepsis, micro-organism isolated from blood cultures, age at follow-up and blinding status of outcome assessors. This review only included infants with sepsis and excluded any cases with suspected sepsis or necrotising enterocolitis. For each study, data from neurodevelopmental assessment tools and relevant NDI definitions were tabulated. For studies with defined dichotomous outcomes as per study NDI definition, the number of infants with NDI in the sepsis and the non-sepsis groups were calculated. In studies where the odds ratio (OR) for association was reported, individual numbers were calculated using the 2 × 2 contingency tables. For individual studies with reported neurodevelopmental outcome assessments as continuous variables, the mean values and standard deviations (SD) for the sepsis and non-sepsis group were recorded. All numbers used were unadjusted.

### 2.4. Assess Risk of Bias in Individual Studies

Each study was assessed for the risk of bias using a modified version of the Cochrane Collaboration’s tool for assessing risk of bias, as seen in Table S2 [11]. Each study was examined for selection bias, performance bias, attrition bias, detection bias and reporting bias and ranked as ‘*low risk*’ or ‘*high risk*’. It was marked ‘*unclear*’ if there was insufficient information to make an assessment.

### 2.5. Data Synthesis and Analysis

STATA version 15 (Stata, College Station, TX, USA) and the STATA meta-analysis software package ‘*metan*’ were used for all statistical analysis. The meta-analysis was conducted using a random-effect model based on the DerSimonian & Laid method [12,13]. Results were reported only from the random-effect model, given the data from the literature was expected to have variable effect size. For studies reporting dichotomous outcome variables (i.e., NDI vs. no NDI), estimates for OR and their corresponding 95% confidence interval (CI) and percentage weight contributing to the overall meta-analysis from each study were calculated. The impact of missing outcome data was explored using a variety of scenarios including available case analysis, best-case scenario, worst-case scenario and the informative missingness model [14]. For studies reporting continuous outcome variables, the mean and SD were compared. Due to the diversity of neurocognitive tools and domains assessed, the results of studies reporting continuous outcome variables were unable to be pooled for meta-analysis.

The inconsistency of effects across studies was measured by estimating statistical heterogeneity across included studies using the I^2^ calculation along with the associated 95% CI [15,16]. The advantage of this measure of inconsistency is that it does not depend on the number of studies and is accompanied by an uncertainty interval—the predictive interval—which shows the predictive distribution of a future trial, based on the extent of heterogeneity [17]. *p* < 0.05 defined statistical significance in the heterogeneity analysis.

To assess the possibility of publication bias, the log transform of the effect size was plotted against the inverse of its standard error to generate a contour-enhanced funnel plot [18]. The plots were visually inspected for asymmetry of data points, which may represent publication bias. Egger’s meta-regression test was performed to examine small study effect to see if the effect decreased with increasing sample size [19]. A subgroup analysis of studies with a follow-up duration of 36 months or greater was performed to investigate long-term NDI.

## 3. Results

### 3.1. Study Selection

**Figure 1:**
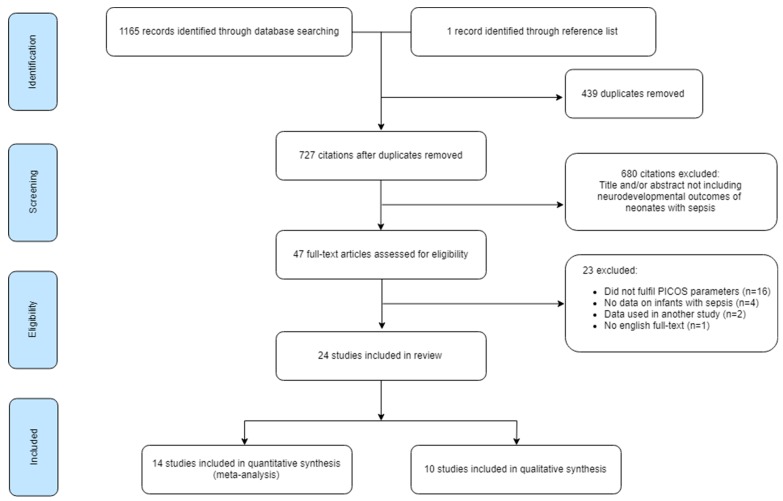
Flow diagram of the study selection process.

The study selection process is summarised in Figure 1. Database searching identified 1165 articles. After removing duplicates, 727 articles were available for screening. A total of 680 were excluded as they did not meet the initial selection criteria. After full-text assessment of the remaining 47 articles, another 23 were excluded [20,21,22,23,24,25,26,27,28,29,30,31,32,33,34,35,36,37,38,39,40,41,42]. The remaining 24 articles were eligible for data analysis and synthesis. Of these, 14 were eligible for quantitative synthesis. The remaining ten studies were analysed via qualitative synthesis. Hand searching through the reference lists of the included articles yielded one additional study which was later excluded during full-text analysis [42].

### 3.2. Study Characteristics

Twenty-four studies met the study selection criteria. The details of the study design and study population are summarised in Table 1. Eight studies were multicentre cohort studies [43,44,45,46,47,48,49,50], 12 were single-centre cohort studies [51,52,53,54,55,56,57,58,59,60,61,62] and four were single-centre case-control studies [63,64,65,66]. All studies were retrospective in design. Ten studies used retrospectively collected data [51,52,53,56,59,60,63,64,65] and 13 studies used prospectively collected data [44,45,46,47,48,49,50,55,57,58,61,62,66]. Publications years were between 1994 and 2019.

Overall, the total number of survivors at follow-up ranged from 33 to 7892, with the number of confirmed sepsis ranging from 13 to 1922 and the number of non-sepsis comparators ranging from 18 to 2161. Birth years were between 1983 and 2014. Overall, the median attrition rate of all studies was 20% (range 3%–45%). Fourteen studies specifically analysed the impact of sepsis [45,46,47,49,52,55,57,58,62,63,64,65,66], whereas the other ten studied perinatal variables more generally with sepsis as a factor [44,48,50,51,53,56,59,60,61]. Fourteen studies did not report the prevalence of each micro-organism [44,47,48,49,50,51,53,54,55,56,59,60,61,66]. Seven analysed the prevalence of each microorganism [43,45,46,58,62,64,65], of which four included fungal infections [43,46,58,65]. Two studies restricted their studies to Candidaemia only [52,63] and one restricted to coagulase-negative *Staphylocci* sepsis only [57]. Follow-up duration ranged from nine to 180 months. Four studies had a follow-up duration of more than or equal to 36 months [47,51,59,60]. Five studies had blinded outcome assessors [49,51,57,58,62], one reported no blinding was performed [54] and the rest did not specify [43,44,45,46,48,50,52,53,55,56,59,60,61,63,64,65,66]. Age of assessment ranged from 18 months to 15 years. All studies contained information on the neurodevelopmental assessment tools used and details of neurodevelopmental outcomes assessed as summarised in Table 2. Nineteen studies reported dichotomised outcomes using a definition for NDI [43,44,45,46,47,48,49,50,51,52,53,54,56,57,59,60,63,65,66], whereas the other five reported outcomes as continuous variables [55,58,61,62,64].

### 3.3. Risk of Bias within Studies

**Figure 2:**
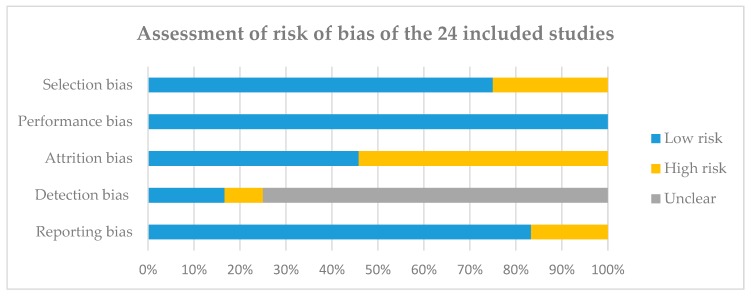
Assessment of risk of bias of the 24 included studies. The risk of bias was assessed using a modified version of the Cochrane Collaboration’s tool for assessing risk of bias; see Table S2.

Figure 2 summarises the risk of bias from all studies; see Table S3 for detail assessment from each study. Overall, six studies were ranked *high risk* and 18 studies were ranked *low risk* for selection bias. All studies were ranked *low risk* for performance bias. Eleven studies were ranked *high risk* and 13 studies were ranked *low risk* for attrition bias. Two studies were ranked *high risk* and four studies were ranked *low risk* for detection bias, with the remaining 18 marked as *unclear*. Four studies were ranked *high risk* and 20 studies were ranked *low risk* for reporting bias.

### 3.4. Quantitative Synthesis

**Figure 3:**
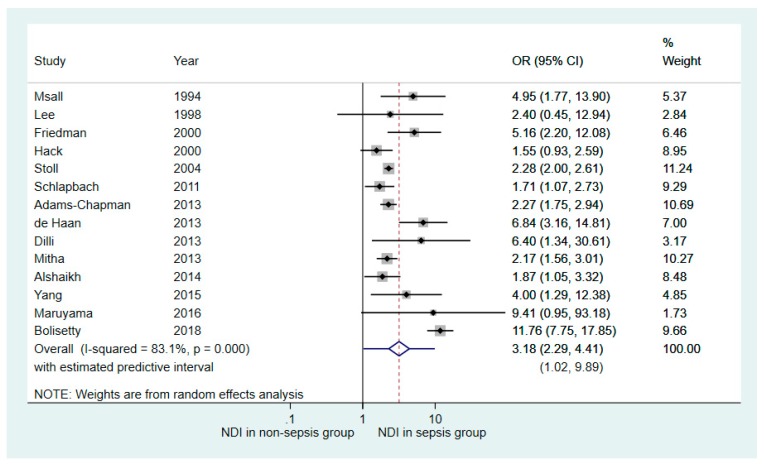
Forest plot showing the results of random effects meta-analysis of the 14 studies comparing neurodevelopmental outcomes in very premature infants with and without neonatal sepsis exposure (Outcome: number of participants with neurodevelopmental impairment). NDI—neurodevelopmental impairment; OR—odds ratio; CI—confidence interval.

Fourteen out of the 19 studies reporting dichotomised neurodevelopmental outcomes contained adequate information for meta-analysis. Table 3 details the number of NDI identified in the sepsis and non-sepsis groups in each study. Overall, 35% of the individuals with culture-proven sepsis had NDI compared with 17% in the non-sepsis group. The median attrition rate in these studies was 18% (range 3%–45%). Figure 3 demonstrates the OR forest plot based on available case analysis and a random effect model. Repeated analyses examining the impact of missing data demonstrated similar outcome trends. The forest plot demonstrated that VP infants with neonatal sepsis had more adverse long-term NDI compared with those without sepsis (OR 3.18, 95% CI 2.29–4.41), as seen in Figure 3. Substantial heterogeneity existed across the studies (I^2^ = 83.1, 95% CI 73–89). The estimated predictive value was 1.02–9.89.

**Figure 4:**
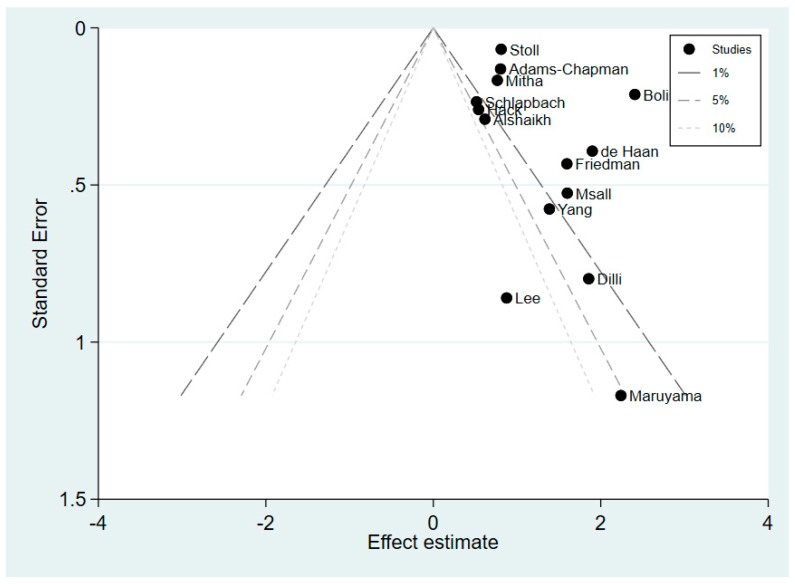
Contour-enhanced funnel plot of the 14 studies with reported dichotomised neurodevelopmental outcomes.

The contour-enhanced funnel plot shown in Figure 4 demonstrates a high likelihood of publication bias, as reflected by high degrees of plot asymmetry with a lack of negative studies. There was a predominance of studies showing statistically significant positive effect. However, on visual assessment of the plot, the publication bias was unlikely due to small study effects. The studies which demonstrated statistically significant findings (i.e., those lying outside the 1% line (*p* < 0.01)) were not restricted to studies with small sample size. This was confirmed quantitatively using the Egger’s meta-regression test. This test demonstrated that smaller studies did not give different results when compared with larger studies as the 95% CI of the intercept did include the zero value (coefficient 1.33, 95% CI −0.76–3.42, *p* = 0.190).

**Figure 5:**
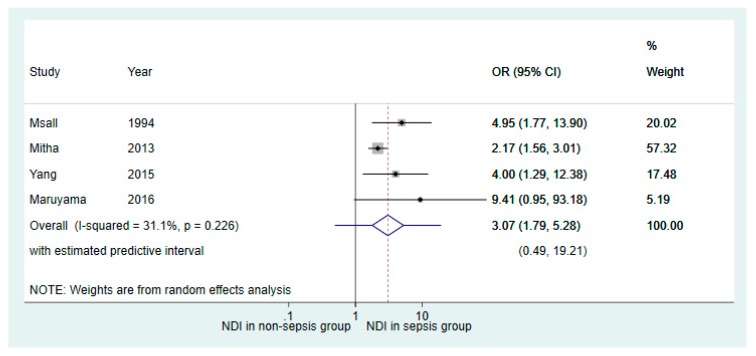
Forest plot of the subanalysis of studies which had a follow-up duration of 36 months or greater. The plot shows the results of random effects meta-analysis of the four studies comparing neurodevelopmental outcomes in very premature infants with and without neonatal sepsis exposure (Outcome: number of participants with neurodevelopmental impairment).

Results of subanalysis based on the four studies with a follow-up duration of 36 months or greater showed similar association between neonatal sepsis and NDI (OR 3.07, 95% CI 1.79–5.28; Figure 5), as compared to the primary meta-analysis conducted from all 14 studies (OR 3.18, 95% CI 2.29–4.41), as seen in Figure 3.

### 3.5. Qualitative Synthesis

The qualitative synthesis included five studies which reported dichotomised outcomes [44,48,49,54,56] but did not have adequate information for meta-analysis, and five studies which reported continuous outcomes [55,58,61,62,64].

Table 3 summarises the five studies which reported dichotomised outcomes. They revealed conflicting findings, as some studies demonstrated no or minimal association between sepsis and NDI [54,56], whilst others showed an association between sepsis and NDI [44,48]. One study showed an association between sepsis and NDI which they argued was attributable to the effect of IQ [49]. Table 4 summarises the five studies which reported continuous outcomes [55,58,61,62,64]. Four of the studies demonstrated no or minimal association [55,58,61,62,64] and only one found significant association between sepsis and cognitive function in the studied VP children [62].

The following sections provide detailed accounts of each study included in this qualitative synthesis.

#### 3.5.1. Studies Reporting Dichotomised Outcomes

Hoekstra et al. [54] retrospectively analysed results from 675 out of 778 (87% follow-up rate) children born at 23–26 weeks gestational age and assessed them between 36 and 60 months of age. The association between sepsis and NDI at a mean age of 47.5 months (range 36–60 months) was not statistically significant. No detailed psychometric testing was done at school age.

Jang et al. [56] retrospectively reviewed medical records of VLBW infants admitted from 1998 to 2007 and compared with those admitted from 1989 to 1997. There was a total of 967 survivors. Univariate analysis showed sepsis was a weak risk factor for cerebral palsy (OR 1.653, 95% CI 0.849–3.215) assessed at 18–24 months. No information was reported on sepsis and noncerebral palsy NDI.

Kono et al. [44] evaluated a prospective cohort of 2847 VLBW survivors. A total of 1826 (64%) completed follow-up at 36–42 months of age. Of the 1826, 113 survivors had neonatal sepsis. An association was found between sepsis and a combined measure of cerebral palsy or death (OR 2.6, 95% CI 1.4–4.8) as well as sepsis and a combined measure of NDI or death (OR 2.8, 95% CI 1.6–4.8). No information was reported between sepsis and NDI only.

Synnes et al. [48] evaluated a prospective national cohort of children born at less than 29 weeks gestational age at 18–21 months. Eighty percent (1870 out of 2340) of the infants completed follow-up. Outcomes were differentiated into NDI and significant NDI based on a prespecified criterion, as seen in Table 2. They found a statistically significant association between sepsis and significant NDI (OR 1.50, 95% CI 1.05–1.86), but no information was reported on association between sepsis and NDI.

Bright et al. [49] reviewed a multicentre prospective study of extremely preterm infants born before 28 weeks gestational age. Ninety-two percent of infants were followed up (889 out of 966) with cognitive assessments at ten years of age, of which 233 infants had neonatal bacteraemia. A wide variety of outcomes were assessed. Confirmed bacteraemia was associated with lower z-scores in outcomes assessing verbal and nonverbal IQ, oral expression, academic achievement, executive function and visual perception. Children with confirmed bacteraemia were more likely to have visual or auditory impairment, but not motor impairment. However, after adjusting for IQ, many of these associations were lost, suggesting IQ may be a mediator between bacteraemia and cognitive deficits.

#### 3.5.2. Studies Reporting Continuous Outcomes

Shah et al. [55], Hentges et al. [58] and Zonnenberg et al. [62] assessed mental development index (MDI) and psychomotor development index (PDI) outcomes of VP infants at 24 months. Shah et al. [55] reported an unadjusted mean difference of 6.8 (95% CI 0.8–12.8, *p* = 0.02) in MDI and 5.6 (95% CI 0.4–10.9, *p* = 0.04) in PDI between VP infants with and without sepsis. After adjusting for potential confounders and white matter abnormalities, group differences decreased and there was no statistical significance, suggesting white matter abnormalities may mediate functional impairment. Hentges et al. [58] and Zonnenberg et al. [62] reported no significant mean difference in MDI and PDI between the two study populations. Zonnenberg et al. [62] found no significant difference in lexical development or behaviour scores between the two groups.

Young et al. [61] reviewed 100 infants born before 32 weeks gestational age at 24 months and 48 months. Using bootstrap ratios of predictors and partial least square regression to draw relationships, they found sepsis was not significantly associated with 2-year or 4-year cognitive measures but was significantly associated with 4-year behavioural measures.

Van der Ree et al. [64] evaluated 50 VP infants (of which 32 had neonatal sepsis) at 6–9 years of age using an extensive list of motor, cognitive and behavioural outcomes. The study demonstrated 68% of the proven sepsis cohort had abnormal or borderline motor skills (OR 3.30, 95% CI 0.98–11.07, *p* < 0.10), particularly poorer fine motor skills (OR 5.46, 95% CI 1.52–1.58, *p* < 0.01). Their total IQ was 89 (14 SD, range 55–118) compared with 98 (8 SD, range 82–110) in VP infants without sepsis, giving an OR of 13.22 (95% CI 1.57–111.75, *p* < 0.05). Verbal memory and attention were significantly affected in those with proven sepsis compared with VP infants without sepsis (0.61 SD, 95% CI 0.04–1.17, *p* = 0.033 and 0.94 SD, 95% CI 0.32–1.62, *p* = 0.011, respectively). There were no significant differences in total behavioural problems between the groups (OR 0.86, 95% CI 0.25–3.00).

## 4. Discussion

This review provides an updated literature synthesis on the long-term neurodevelopmental impact of neonatal sepsis in VP infants compared to those without sepsis. The conducted meta-analysis demonstrated statistically significant association between sepsis and NDI, but was limited by the substantial heterogeneity that existed between studies. On the contrary, qualitative synthesis of the remaining studies revealed less clear associations between sepsis and long-term neurocognitive deficits. There were limited studies examining continuous outcomes of NDI after sepsis. The included studies in this review offer valuable insight, but will need to be interpreted in the context of the study limitations.

### 4.1. Study Designs

All studies were observational studies, as randomised control trials were not ethically possible given the nature of the topic. In case-control studies, controls were generally matched for admission period, gestational age and/or birth weight. These studies may have suffered from selection bias and reporting bias. One case-control study reported they selected controls by randomly selecting every fifth subject admitted to the NICU within a specific time-frame [65]. Case-cohort studies used either retrospectively collected data or prospectively collected data. Prospective data collection is dependent on the proportion of infants successfully followed up which determines attrition bias. Retrospective data collection can be limited as not all information for the study may be collected at the time, which can result in reporting bias. In addition, all retrospective reviews rely on accurate data entries and recordings.

In retrospective studies, increasing sample size results in increasing statistical power. Some studies included in this review had much smaller study populations compared with other studies (i.e., the sample size in all included studies ranged from 33 to 7892). This would contribute to the overall statistical heterogeneity and impede the validity and interpretation of study outcome. Adams-Chapman et al. [46] reported that the non-sepsis group in the *Candida* study was not representative of the homogeneous extremely low birthweight population and thus performed a secondary analysis using additional non-sepsis infants enrolled in the registry by the Neonatal Research Network (NRN) of the *Eunice Kennedy Shriver* National Institute of Child Health and Health Development (NICHD). Although this increases statistical power, it also increases the risk of selection and reporting bias.

Similarly, several of the included studies consisted of disproportionate sample size in the sepsis versus the non-sepsis group, with up to ten times greater numbers in the non-sepsis group compared to the sepsis group in one study [51,52]. For example, Msall et al. [51] investigated sepsis as a perinatal factor and of the 149 participants followed up, only 18 had confirmed sepsis. Van der Ree et al. [64] compared 32 infants with sepsis to 18 non-sepsis infants. Due to the disparity in group numbers, with much smaller numbers in the sepsis group, there is a higher chance of over-representing infants developing NDI in the sepsis group as compared to the much larger non-sepsis group. This overestimation can result in publication bias as studies are more inclined to publish results with significant findings.

### 4.2. Population

All studies adjusted for selected confounding factors such as age and sex. However, they adjusted differently with various combinations of other factors such as multiple birth, mode of delivery, corticosteroid exposure, intrauterine growth restriction, bronchopulmonary dysplasia, necrotising enterocolitis, chorioamnionitis, periventricular leukomalacia, socioeconomic status and maternal education. As a result, we extracted unadjusted numerical data to pool, but were unable to assess the impact of these confounding factors.

The median attrition rate was 20% and the range was wide (3%–45%). Loss through follow-up can be due to many factors, such as unable to be contacted, parent refusal, or being too impaired to participate in tests [67]. Most studies did not report the reason for loss to follow-up. Some studies compared patient demographics between infants who completed follow-up with those who did not to identify attrition bias. As loss to follow-up increases, bias in the reported outcome also increases [68]. Prevention of attrition bias is inherently difficult and best to be addressed in prospectively designed studies.

One potential confounder may be related to the wide range in birth years of the infants, and different ages at assessment. For instance, infants born in earlier eras may have received less advanced neonatal care as compared to more recent eras. Furthermore, quality of neonatal care may vary across regions and countries, potentially influencing the events experienced by the infant during the neonatal period and the subsequent neurodevelopmental outcomes.

### 4.3. Assessment of Sepsis

One limitation of this review was the definition of neonatal sepsis as an appreciable portion of neonatal sepsis is blood culture-negative or have positive cultures from other sterile sites. For a focused review, we defined sepsis as having a blood culture-proven infection which has historically been the ‘gold standard’, but there was great variability amongst studies [4,69]. Definitions included: positive blood culture only [44,46,48,49,52,54,56,59,60,62,63,64,66]; positive blood culture and clinical signs of sepsis [50,53,58,65]; positive blood culture and antibiotic use [43,47,51,61]; positive blood culture, clinical signs of sepsis and antibiotic use [57]; positive blood culture, antibiotic use and presence of raised inflammatory markers [55]; and positive blood culture, clinical signs of sepsis, antibiotic use and presence of raised inflammatory markers [45]. Other specific variations include time that the blood culture was taken, duration of antibiotic use and type of infection laboratory markers examined. Some of these studies justified that using a blood culture only definition has suboptimal sensitivity and specificity, as there is risk of contamination which could produce false positives [55]. Infants who had clinically suspicious sepsis, but no positive blood culture may also be missed. The diversity in sepsis definitions could contribute to the population heterogeneity and overall selection bias.

Selected studies that analysed the impact of specific micro-organisms may also contribute to selection bias. Lee et al. [63], Friedman et al. [52], and Adams-Chapman et al. [46] focused on Candidaemia, De Haan et al. [65] examined Candidaemia and Gram-negative organisms, whereas Alshaikh et al. [57] investigated coagulase-negative *Staphylocci* specifically. In contrast, Stoll et al. [43], Schlapbach et al. [45], and Hentges et al. [58] did a micro-organism subtype analysis and reported the association between predominant organisms and outcomes. For instance, Hentges et al. [58] found no statistical difference between the two study groups, but upon further analysis, reported VLBW infants with Gram-positive sepsis showed higher rates of motor deficit when compared to the non-sepsis group (68.8% and 29.3% respectively). Similarly, Schlapbech et al. [45] did a subtype analysis and found infants who had Gram-positive sepsis had the poorest outcomes. This suggests different organisms may cause different deficits which should be further explored in future studies.

### 4.4. Assessment of Outcomes

Functional outcomes must be interpreted carefully given the variability between studies. Most studies focused on early outcomes, with half of the included studies in this review performing assessments at or before the age of 24 months. Only five studies examined outcomes beyond 5 years of age [47,49,51,59,64], two of which investigated outcomes beyond the age of ten [49,59]. No studies assessed outcomes in early adulthood or reported if difficulties persisted into early adulthood. Cognitive skills are complex and may evolve overtime with brain maturation. Early assessments may underestimate the full spectrum of outcomes, such as milder motor dysfunctions and specific learning disorders, and be of less predictive value for long-term outcomes [45,66]. Most studies performed a single cross-sectional follow-up, but outcomes may alter over time. Children who test within the normal range at early follow-up may still remain at risk of developing significant problems later in life [43,54,57]. Conversely, children with early deficits may improve or remain unchanged.

Eighteen out of the 24 included studies did not specify if outcome assessors were blinded to the infants’ neonatal history [43,44,45,46,48,50,52,53,55,56,59,60,61,63,64,65,66]. Five studies reported blinding of outcome assessors [49,51,57,58,62] and one reported no blinding was performed [54]. Without blinding, there is a high chance of detection and reporting bias as assessors may perceive sepsis-affected infants to be at higher risk of functional deficits. Although the tests are standardised, most studies did not note the experience level of outcome assessors, which may also contribute to the risk of detection and reporting bias.

Two approaches were used to assess outcomes: using dichotomised (using a prespecified NDI definition) or continuous (calculating mean and SD of groups) outcomes. In the former, variation in NDI definition was a significant limitation. Most studies defined NDI as cerebral palsy or its components, cognitive and psychomotor scores of <70 or more than two SD below the mean, visual impairment or hearing impairment. Lee et al. [63] defined cognitive delay as more than three SD below the mean which would exclude children who scored between two and three SD below the mean. In contrast, Yang et al. [59] utilised a different definition whereby the parent had to disclose that the child was ‘*disabled*’, a handicap status issued by the Ministry of the Interior of Taiwan, which is a subjective evaluation of disability. Fifteen studies used BSID-II as an assessment tool where MDI and PDI was defined as a score of <70 or more than two SD below the mean. BSID-II is useful for infants who are yet to enter preschool as it provides an indicator if the infant is at risk of developmental delay, therefore the caregiver has the opportunity to seek early intervention to improve outcomes. The 2005 BSID revision saw a shift from BSID-II to Bayley-III. Four studies used Bayley-III for assessment [46,48,50,61]. However, studies have reported that the Bayley-III detects lower rates of NDI in infants as compared to BSID-II using the same population [70,71,72]. There is also limited data on the long-term predictive validity of Bayley-III [72]. Kono et al. [44], De Haan et al. [65] and Maruyama et al. [60] looked at NDI and death together, which may overestimate the study effect of sepsis in VP infants. Overall, dichotomised studies tended to focus on identifying the most impaired children, often at an early age. This was reflected by the reporting of incomplete assessments in several studies due to reasons such as distractions or limitations in the child’s ability to complete the assessment.

Accordingly, less focus was placed on less impaired children who may have more subtle cognitive difficulties. To address this, studies using continuous outcomes can identify subtle impairments as a child grows older and begins school. For instance, Van der Ree et al. [64] noted that out of the 18 children with neonatal sepsis exposure who had normal full-scale IQs, nine of them had problems in attention or memory. Limited studies (*n* = 5) reported continuous outcome variables, and the heterogeneity of the reporting cognitive domains in each study made it difficult to pool these results. In these studies, the mean results were comparable between the sepsis group and non-sepsis group. One potential factor contributing to the conflicting results may be the Zonnenberg et al. [62] study, as they compared proven sepsis versus no proven sepsis in a cohort that all had an episode of suspicious infection, which questions the incorporation of clinically suspicious infants into our analysis and introduces selection bias. Only one study used a wide variety of assessments in a continuous manner to extensively study the neurocognitive deficits a child may be experiencing [64].

### 4.5. Review Limitations

This review has several limitations. Data screening, extraction, and analysis were performed by one reviewer (S.C.), instead of consensus reached by at least two independent reviewers. However, data synthesis was discussed with, and consensus was reached with the senior author (J.Y.-M.Y). The reviewer did not attempt to contact the included study authors for missing data. Thus, this systematic review did not strictly adhere to the recommended Preferred Reporting Items for Systematic Reviews and Meta-analyses (PRISMA) guidelines [73]. Secondly, sensitivity analyses were not performed to examine the influence of attrition bias and the contribution of different micro-organisms. Given that substantial disagreement remains in the definition of sepsis, selecting a sepsis definition also subjects our review to this methodological limitation. Our review did not address the impact of neonatal meningitis and urinary tract infection separately, which are both common in the NICU setting.

### 4.6. Recommendations for Future Research

Based on the results of this systematic review, we recommend future studies examining cognitive outcomes of VP infants with neonatal sepsis. Longitudinal studies of prospectively collected data would be the most appropriate study design. Larger cohorts and high rates of follow-up would increase statistical power and reduce attrition bias. If follow-up rates are low, assessment of the characteristics between infants who do complete follow-up and those who do not would be needed. There is much evidence to support the risk of major NDI, but little research has been done in identifying more subtle difficulties in less impaired children which may be significant during activities of daily living. To dissect the specific cognitive impairments, a range of assessment tools should be used to measure motor function, cognitive skills and behaviour as continuous variables. Participants should be assessed later in life, ideally once they have entered the education curriculum, as there is a higher predictive value in dissecting long-term outcomes.

## 5. Conclusions

Neonatal sepsis can have a profound impact on neurodevelopment of VP infants. This systematic review found 24 published studies and performed meta-analysis in 14 studies using prespecified NDI definitions. This review suggests that the most impaired VP infants surviving neonatal sepsis may be at higher risk for long-term neurodevelopmental disability compared with VP infants without sepsis. However, current evidence is limited by significant statistical heterogeneity and publication bias due to significant differences in the included study design and definitions used for NDI. Moreover, this finding could not be extrapolated to all VP infants surviving neonatal sepsis due to lack of studies reporting cognitive outcomes using non-discrete, continuous variables. Current published studies lack long-term longitudinal follow-up data. These results highlight the necessity for future longitudinal studies to use continuous outcomes performed at a later age to discern the subtle and more specific long-term cognitive risks for VP children with neonatal sepsis.

## Figures and Tables

**Table 1:** Summary of the study design and study population characteristics of all included studies.

Author	Year	Study Design	Population	Birth Year	No. of Survivors at Follow-Up	Follow-Up Rate	Total No. Followed Up	No. of Confirmed Sepsis	No. of Non-sepsis Comparators	Organism Isolated	Age at Assessment (Months)	Blinding of Outcome Assessors
**Msall [51]**	1994	SC, cohort	GA 23–28	1983–1986	153	97%	149	18	131	NS	52–62	Yes
**Lee [63]**	1998	SC, case control	BW < 1250	1990–1995	35	N/A	N/A	14	21	Candida	9–50	NS
**Friedman [52]**	2000	SC, cohort	ELBW	1988–1996	334	90%	299	27	272	Candida	21–24	NS
**Hack [53]**	2000	SC, cohort	ELBW	1992–1995	241	92%	221	109	112	NS	20	NS
**Hoekstra [54]**	2004	SC, cohort	GA 23–26	1986–2000	778	87%	675	NS	NS	NS	36–60	No
**Stoll [43]**	2004	MC, cohort	ELBW	1993–2001	7892	80%	6314	1922	2161	Shows breakdown	18–22	NS
**Shah [55]**	2008	SC, cohort	GA < 30	2001–2003	204	94%	192	64	119	NS	24	NS
**Kono [44]**	2011	MC, cohort	VLBW	2003–2004	2847	64%	1826	113	1714	NS	36–42	NS
**Jang [56]**	2011	SC, cohort	VLBW	1989–2007	967	N/A	N/A	NS	NS	NS	18–24	NS
**Schlapbach [45]**	2011	MC, cohort	GA 24–27	2000–2007	482	77%	372	136	236	Shows breakdown	18–24	NS
**Van der Ree [64]**	2011	SC, case-control	GA < 32 or VLBW	2000–2001	50	N/A	N/A	32	18	Shows breakdown	72–108	NS
**Adams-Chapman [46]**	2013	MC, cohort	ELBW	2004–2007	1966	71%	1391	474	917	Candida and other	18–22	NS
**De Haan [65]**	2013	SC, case-control	GA < 30	1997–2007	168	90%	152	50	102	GN and Candida	24	NS
**Dilli [66]**	2013	SC, case control	VLBW	2008–2009	33	N/A	N/A	13	20	NS	18–24	NS
**Mitha [47]**	2013	MC, cohort	GA 22–32	1997	2277	78%	1769	688	1081	NS	60	NS
**Alshaikh [57]**	2014	SC, cohort	GA < 29	1995–2008	383	87%	332	105	227	CONS	30–42	Yes
**Hentges [58]**	2014	SC, cohort	GA < 32 or VLBW	2003–2010	291	78%	226	62	164	Shows breakdown	18–24	Yes
**Yang [59]**	2015	SC, cohort	VLBW	1996–1999	111	55%	61	26	35	NS	144–180	NS
**Maruyama [60]**	2016	SC, cohort	VLBW	2005–2012	200	78%	155	N/A	N/A	NS	36–42	NS
**Synnes [48]**	2016	MC, cohort	GA < 29	2009–2011	2340	80%	1870	NS	NS	NS	18–21	NS
**Young [61]**	2016	SC, cohort	GA < 32	2008–2010	100	N/A	N/A	17	33	NS	24, 48	NS
**Bright [49]**	2017	MC, cohort	GA < 28	2002–2004	966	92%	889	223	532	NS	120	Yes
**Bolisetty [50]**	2018	MC, cohort	GA 23–28	2007–2012	1897	80%	1514	526	988	NS	24–36	NS
**Zonnenberg [62]**	2019	SC, cohort	GA < 32 or VLBW	2008–2014	104	87%	90	68	22	Shows breakdown	24	Yes

Abbreviations: SC—single-centre, MC—multicentre, GA—gestational age (weeks), BW—birth weight (grams), ELBW—extremely low birth weight (<1000 g), VLBW—very low birth weight (<1500 g), GN—Gram-negative organisms, CONS—coagulase negative *Staphylococci*, N/A—not applicable, NS—not specified.

**Table 2:** Neurodevelopmental outcome scales used by the included studies and their respective neurodevelopmental impairment definitions.

Study	Scale Used	NDI Definitions
**Studies Which Reported Dichotomised Outcomes**		
Msall [51]	McCarthy Scales of Children’s Abilities, Cattell Infant Intelligence Test or the Clinical Linguistic Auditory Milestone Scale (for children with a mental age < 30 months)	One or more of the following: - CP defined as early onset, nonprogressive motor and postural delay (hemiplegia, diplegia or quadriplegia) - IQ: Mild 52–67, Moderate 36–51, Severe 20–35 on McCarthy instrument - Blindness defined as legal blindness or corrected vision worse than 20/200 - Deafness defined as bilateral hearing impairment of more than 85 dB
Lee [63]	BSID-II (<24 months), Stanford–Binet Intelligence Scale, Peabody Developmental Motor Scales	One or more of the following: - CP of all types of severity - Legal blindness (corrected visual acuity of the better eye, <20/200) - Hearing loss (neurosensory hearing loss in the better ear, >30 dB) - Cognitive delay (MDI, >3 SD below the mean)
Friedman [52]	BSID-II	Mild-moderate: mild-moderate CP, moderate cognitive delay (MDI 70–82) Severe: severe CP (not sitting by 2 years of age, non-ambulatory), bilateral blindness, aided sensorineural hearing loss, severe cognitive delay (MDI < 70), shunted hydrocephalus
Hack [53]	BSID-II	One or more of the following: - CP (spastic diplegia, hemiplegia, hemiplegia, or quadriplegia), hypertonia, hypotonia and shunt-dependent hydrocephalus - Blindness unilateral or bilateral - Deafness unilateral or bilateral - MDI < 70
Hoekstra [54]	BSID-II (<36 mo)	Mild–moderate: isolated muscle tone abnormalities, unilateral blindness, hyperactivity, scores 1–2 SD below mean Severe: spasticity, severe hypotonia, blindness, deafness, scores >2 SD below mean
	3–6 years: Denver Developmental Screening Test, Early Language Milestone Scale, Zimmerman Preschool Articulation Test	Mild–moderate: minor abnormalities, developmental assessments 6–12 months below chronological age Severe: severe physical and neurologic examinations, developmental assessments >1 year below chronological age
	School children: University of Vermont Achenbach Child Behaviour Checklist and the Teacher’s Report form	Mild–moderate: below grade average in >1 subject, but not far below in >1 subject, or had below-normal Achenbach scores Severe: spasticity, severe hypotonia, blindness, hearing loss, repeated a grade, require special education, far below grade average in >1 subject or Achenbach scores far below normal
Stoll [43]	BSID-II	One or more of the following: - CP - MDI < 70 or PDI < 70 - Bilateral blindness - Bilateral hearing impairment
Kono [44]	KPSD	One or more of the following: - CP (nonprogressive CNS disorder characterised by abnormal muscle tone in at least one extremity and abnormal control of movement and posture) - Unilateral or bilateral blindness - Hearing impairment requiring hearing aids - KPSD < 70 or judged by physicians (i.e., ability to say any meaningful words, ability to say own name or age, able to build using several small bricks, able to distinguish size of circles with a diameter of 4 cm and 6 cm)
Jang [56]	BSID or Denver Developmental Screening Test	One or more of the following: - CP (permanent, but no unchanging, disorder of movement and/or posture and of motor function caused by a nonprogressive interference, lesion, or abnormality of the developing immature brain) - >6 months delay of motor and/or mental development including cognitive impairment, psychomotor impairment and neurosensory impairment
Schlapbach [45]	BSID-II, GMFCS	One or more of the following: - CP (nonprogressive motor disorder characterised by abnormal tone in at least one extremity and abnormal control of movement and posture) - MDI < 70 or PDI < 70 - Bilateral blindness - Hearing impairment requiring amplification
Adams-Chapman [46]	BSID-II, BSID-III, GMFCS	BSID-II (Epoch 1) - Moderate–severe CP with GMFCS ≥ 2 - MDI < 70 or PDI < 70 - Bilateral blindness with no functional vision - Bilateral amplification for permanent hearing loss BSID-III (Epoch 2) - Moderate to severe CP with GMFCS ≥ 2 - BSID-III cognitive < 70 - Visual acuity < 20/200 bilateral - Permanent hearing loss that does not permit the child to understand directions of examiner and communicate despite amplification
De Haan [65]	BSID-II-NL	One or more of the following: - CP ± clinical hearing loss or visual handicaps - MDI < 85 or PDI < 85
Dilli [66]	BSID-II, GMFCS	One or more of the following: - Moderate–severe CP - MDI < 70, PDI < 70 - Bilateral deafness - Bilateral blindness
Mitha [47]	Kaufman Assessment Battery for Children	- CP (at least two of the following: abnormal posture or movement, increased tone, hyperreflexia) - MPC < 70
Alshaikh [57]	BSID-II, WPPSI-Revised, Stanford–Binet IV	One or more of the following: - CP (non-progressive motor impairment characterised by abnormal muscle tone in at least one extremity and decreased range or control of movements) - Cognitive delay >2 SD below mean on standardised assessment (WPPSI-Revised, BSID-II or Stanford–Binet IV) - Sensorineural hearing loss requiring amplification - Visual acuity <20/200 following refractive correction
Yang [59]	CBCL, “Current Status Survey”, WISC-IV, MINI-KID (for ADHD, anxiety/mood disorders), DSM-IV-TR (for ASD)	“Disabled” = when parents disclosed that the child received a handicap status as issued by the Ministry of the Interior of Taiwan. Handicap is defined as disadvantaged condition, deriving from impairment or disability limiting a person performing a role considered normal in respect of their age, sex and social and cultural factors.
Maruyama [60]	KPSD	One or more of the following: - CP - Unilateral or bilateral blindness - Severe hearing impairment - Developmental delay: DQ < 70
Synnes [48]	BSID-III, GMFCS	Severe NDI - CP with GMFCS III, IV or V - Bayley-III motor composite < 70, cognitive composite < 70, language composite < 70 - Hearing aid or cochlear implant - Bilateral visual impairment NDI - CP with GMFCS I - Bayley-III motor composite < 85, cognitive < 85, language composite < 85 - Sensorineural/mixed hearing loss - Unilateral or bilateral visual impairment
Bright [49]	GMFCS, DAS-II, OWLS, NEPSY-II, WIAT-III, Manual Ability Classification Test	- Parent-reported legally blind—severe visual impairment - Severe auditory impairment—parent-reported child has hearing aids or cochlear plant and/or receives special services for the hearing-impaired
Bolisetty [50]	BSID-III, GMFCS	Moderate - BSID-III 2–3 SD below mean - Moderate CP GMFCS level 2 or 3 (able to walk with the assistance of aids) - Bilateral deafness (requiring amplification with hearing aids or unilateral/bilateral cochlear implants) Severe - BSID-III >3 SD below mean - Severe CP GMFCS level 4 or 5 (unable to walk with the assistance of aids) - Bilateral blindness (visual acuity of <6/60 in better eye)
**Studies Which Reported Continuous Outcomes**		
Shah [55]	BSID-II	N/A
Van der Ree [64]	Bax’ criteria, GMFCS, Movement ABC, WISC-III-NL, NEPSY-II, AVLT, TEA-Ch, ADHD questionnaire, BRIEF, CBCL	N/A
Hentges [58]	BSID-II	N/A
Young [61]	At 2 years: BSID-III At 4 years: WPPI-III, CLEF-2, Beery-Buktenica Test of Visual Motor Integration, the Behaviour Assessment System for Children Parent Rating Scales, Behavioural Rating Inventory of Executive Functioning-Preschool	N/A
Zonnenberg [62]	BSID-II, Lexilijst (lexical development questionnaire), CBCL	N/A

Abbreviations: NDI—neurodevelopmental impairment; CP—cerebral palsy; BSID-II—Bayley Scales of Infant Development, Second Edition; MDI—Mental Development Index; PDI—Psychomotor Development Index; KPSD—Kyoto Scale of Psychological Development; DQ—Development Quotient; GMFCS—Gross Motor Function Classification System; Bayley-III—Bayley Scales of Infant and Toddler Development, Third Edition; MPC—Mental Processing Composite score; WPPSI-Revised—Wechsler Preschool and Primary Scales of Intelligence, Revised; CBCL—Child Behaviour Checklist; WISC-IV—Wechsler Intelligence Scale for Children-IV; MINI-KID—Mini-International Neuropsychiatric Interview for Children and Adolescents; DAS-II—School-Age Differential Ability Scales, Second Edition; OWLS—Oral Written Language Scales; NEPSY-II—Neuropsychological Assessment, Second Edition; WIAT-III—Wechsler Individual Achievement Test, Third Edition; M-ABC—Movement Assessment Battery for Children; WISC-III—Wechsler Intelligence Scale for Children, Third edition; AVLT—Rey’s Auditory Verbal Learning Test; TEA-Ch—Test of Everyday Attention for Children; BRIEF—Behavior Rating Inventory of Executive Function; WPPSI-III—Wechsler Preschool and Primary Scales of Intelligence, Third edition; CELF-2—Clinical Evaluation of Language Fundamentals—Preschool, Second Edition; WASI—Wechsler Abbreviated Scale of Intelligence; M-ABC2—Movement Assessment Battery for Children, Second Edition; CELF-4—Clinical Evaluation of Language Fundamentals, Fourth Edition; CLI—Core Language Index; WMTB-C—Working Memory Test Battery for Children; SD—standard deviation(s); N/A—not applicable.

**Table 3:** Summary of the 19 studies with reported dichotomised neurodevelopmental outcomes.

	Number of Infants with NDI			
Study	Sepsis Group	No Sepsis Group	Attrition Rate	Comments
**Msall [51]**	9/18 (50%)	22/131 (17%)	3%	N/A
**Lee [63]**	4/14 (29%)	3/21 (14%)	N/A	N/A
**Friedman [52]**	11/27 (41%)	32/272 (12%)	10%	N/A
**Hack [53]**	43/93 (46%)	62/112 (55%)	8%	N/A
**Hoekstra [54]** #	NS	NS	13%	At 47.5 months (range 36–60), there was no statistically significant association between primary or secondary sepsis and NDI.
**Stoll [43]**	861/1778 (48%)	576/1976 (29%)	20%	N/A
**Kono [44]** #	NS	NS	36%	At 36–42 months, there was an association between sepsis and cerebral palsy or death (OR 2.6, 95% CI 1.4–4.8) as well as sepsis and NDI or death (OR 2.8, 95% CI 1.6–4.8).
**Jang [56]** #	NS	NS	N/A	At 18–24 months, univariate analysis showed weak association between sepsis and cerebral palsy (OR 1.653, 95% CI 0.849–3.215).
**Schlapbach [45]**	46/134 (34%)	55/235 (23%)	23%	N/A
**Adams-Chapman [46]**	148/474 (31%)	153/917 (17%)	16%	N/A
**De Haan [65]**	28/50 (56%)	16/102 (17%)	10%	N/A
**Dilli [66]**	8/13 (62%)	4/20 (20%)	N/A	N/A
**Mitha [47]**	84/643 (14%)	73/1126 (6%)	22%	N/A
**Alshaikh [57]**	26/105 (25%)	34/227 (15%)	13%	N/A
**Yang [59]**	13/26 (50%)	7/35 (20%)	45%	N/A
**Maruyama [60]**	3/4 (75%)	37/153 (24%)	28%	N/A
**Synnes [48]** #	NS	NS	20%	At 18–21 months, there was significant association between sepsis and significant NDI (OR 1.50, 95% CI 1.05–1.86), but no information was reported on association between sepsis and NDI.
**Bright [49]** #	NS	NS	8%	At 10 years of age, children who had confirmed bacteraemia were associated with lower z-scores in verbal and nonverbal IQ, oral expression, academic achievement, executive function and visual impairment. They were also more likely to have visual and auditory impairment but not motor deficits. After adjusting for IQ, many of these associations were lost.
**Bolisetty [50]**	138/526 (26%)	29/988 (3%)	20%	N/A
**Total**	1422/3905 (36%)	1103/6315 (17%)	Median: 18%	

#: studies did not report absolute numbers for NDI for each study group. NS—not specified; N/A—not applicable.

**Table 4:** Summary of the five studies with reported continuous neurodevelopmental outcomes.

Study	Assessment Tool	Sepsis Group	Non-sepsis Group	Attrition Rate	Comments
		Mean (SD)	Mean (SD)		
Shah [55]	MDI (BSID-II)	79.7 (21.2)	86.5 (18.7)	6%	N/A
	PDI (BSID-II)	84.2 (21.4)	89.9 (14.5)		
Van der Ree [64]	Movement ABC Total (M-ABC2)—raw score	13 (10)	8 (6)	N/A	N/A
	Total intelligence (WISC-II)—IQ	89 (13)	98 (8)		
	Total behavioural problems (CBCL)—t-scores	53 (12)	56 (9)		
Hentges [58]	MDI (BSID-II)	85.9 (10.8)	86.1 (11.59)	22%	N/A
	PDI (BSID-II)	89.8 (13.3)	91.7 (14.02)		
Young [61] #	At 2 years: BSID-III	NS	NS	N/A	Sepsis was not significant with 2-year or 4-year cognitive measures, but significant with 4-year behavioural measures.
	At 4 years: WPPI-III, CLEF-2, Beery–Buktenica Test of Visual Motor Integration, the Behaviour Assessment System for Children Parent Rating Scales, Behavioural Rating Inventory of Executive Functioning – Preschool				
Zonnenberg [62]	MDI (BSID-II)	100 (9.0)	98 (13.9)	23%	N/A
	PDI (BSID-II)	100 (9.4)	99 (12.3)		
	Lexiquotient (Lexilijst)	91 (16.1)	88 (18.2)		
	Total behavioural score (CBCL)	26 (14.9)	30 (21.2)		
	Total internalising score (CBCL)	5 (4.3)	8 (7.9)		
	Total externalising score (CBCL)	12 (7.5)	12 (7.6)		
				Median: 22%	

#: Studies did not report group mean and SD for sepsis and non-sepsis group. NS—not specified.

## Supplementary Materials

The following are available online at https://www.mdpi.com/2227-9067/6/12/131/s1, Table S1. Detailed literature search strategy, Table S2. The Cochrane Collaboration’s tool for assessing risk of bias, Table S3. Assessment of risk of bias of the 24 included studies.
