# Supplementary material for: Short- and Long-Term Neurodevelopmental Outcomes of Very Preterm Infants with Neonatal Sepsis: A Systematic Review and Meta-Analysis
Source: Children (Basel). 2019 Dec 1;6(12):131. doi: 10.3390/children6120131 (PMC6956113; doi:10.3390/children6120131)
Supplement: Supplementary file 1 [file children-06-00131-s001.pdf]

Table S1. Detailed literature search strategy

**Medline**	1. exp infant, low birth weight/ or exp infant, premature/<br>2. (prematur* or pre-matur* or pre-term or preterm or (low adj birth adj weight) or LBW or VLBW or ELBW).tw,kf.<br>3. exp Sepsis/<br>4. (sepsis or sepses or septic?emia* or septic-shock or bacter?emia* or candidiasis or candid?emia*).tw,kf.<br>5. neurodevelopmental disorders/ or developmental disabilities/ or intellectual disability/ or learning disorders/ or motor skills disorders/<br>6. (neurodevelopment* or neuro-development* or long-term-outcome*).tw,kf.<br>7. (1 or 2) and (3 or 4) and (5 or 6)
**Embase**	1. prematurity/<br>2. exp low birth weight/<br>3. exp very low birth weight/<br>4. (prematur* or pre-matur* or pre-term or preterm or (low adj birth adj weight) or LBW or VLBW or ELBW).tw,kw,dq.<br>5. exp sepsis/<br>6. exp fungemia/<br>7. (sepsis or sepses or septic?emia* or septic-shock or bacter?emia* or candidiasis or candid?emia*).tw,kw,dq.<br>8. nerve cell differentiation/<br>9. exp developmental disorder/<br>10. (neurodevelopment* or neuro-development* or long-term-outcome*).tw,kw,dq.<br>11. (1 or 2 or 3 or 4) and (5 or 6 or 7) and (8 or 9 or 10)
**Pubmed**	(prematur* OR pre-matur* OR pre-term* OR preterm* OR low-birth-weight OR low-birthweight OR LBW OR VLBW OR ELBW) AND (sepsis OR sepses OR septicaemia OR septicaemia OR septic OR septic-shock OR bacteraemia OR bacteremia OR candidiasis OR candidaemia OR candidemia OR newborn-sepsis OR newborne-sepsis OR neonatal-sepsis) AND (neurodevelopment* OR neuro-development* OR development* OR developmental-disabilit*) AND (NOTNLM OR publisher[sb] OR inprocess[sb] OR pubmednotmedline[sb] OR indatareview[sb] OR pubstatusaheadofprint)

Table S2. The Cochrane Collaboration's tool for assessing risk of bias

**Domain**	**Support for judgement**	**Review authors' judgement**
Selection bias		
**Random sequence generation**	Describe the method used to generate the allocation sequence in sufficient detail to allow an assessment of whether it should produce comparable groups.	Selection bias (biased allocation to interventions) due to inadequate generation of a randomised sequence.
**Allocation concealment**	Describe the method used to conceal the allocation sequence in sufficient detail to determine whether intervention allocations could have been foreseen in advance of, or during, enrolment.	Selection bias (biased allocation to interventions) due to inadequate concealment of allocations prior to assignment.
Performance bias		
**Blinding of participants and personnel**<br><br>*Assessments should be made for each main outcome (or class of outcomes).*	Describe all measures used, if any, to blind study participants and personnel from knowledge of which intervention a participant received. Provide any information relating to whether the intended blinding was effective.	Performance bias due to knowledge of the allocated interventions by participants and personnel during the study.
Detection bias		
**Blinding of outcome assessment**<br><br>*Assessments should be made for each main outcome (or class of outcomes).*	Describe all measures used, if any, to blind outcome assessors from knowledge of which intervention a participant received. Provide any information relating to whether the intended blinding was effective.	Detection bias due to knowledge of the allocated interventions by outcome assessors.
Attrition bias		
**Incomplete outcome data**<br><br>*Assessments should be made for each main outcome (or class of outcomes).*	Describe the completeness of outcome data for each main outcome, including attrition and exclusions from the analysis. State whether attrition and exclusions were reported, the numbers in each intervention group (compared with total randomized participants), reasons for attrition/exclusions where reported, and any re-inclusions in analyses performed by the review authors.	Attrition bias due to amount, nature or handling of incomplete outcome data.
Reporting bias		
**Selective reporting**	State how the possibility of selective outcome reporting was examined by the review authors, and what was found.	Reporting bias due to selective outcome reporting.
Other bias		
**Other sources of bias**	State any important concerns about bias not addressed in the other domains in the tool.<br><br>If particular questions/entries were pre-specified in the review's protocol, responses should be provided for each question/entry.	Bias due to problems not covered elsewhere in the table.

Adapted from Higgins et al.[11]

Table S3. Assessment of risk of bias of the 24 included studies.

Study	Year	Selection bias	Performance bias	Attrition bias	Detection bias	Reporting bias
**Msall[51]**	1994	High risk	Low risk	Low risk	High risk	High risk
**Lee[63]**	1998	High risk	Low risk	Low risk	Unclear	Low risk
**Friedman[52]**	2000	Low risk	Low risk	High risk	Unclear	Low risk
**Hack[53]**	2000	Low risk	Low risk	Low risk	Unclear	Low risk
**Hoekstra[54]**	2004	Low risk	Low risk	Low risk	High risk	Low risk
**Stoll[43]**	2004	Low risk	Low risk	High risk	Unclear	Low risk
**Shah[55]**	2008	Low risk	Low risk	Low risk	Unclear	Low risk
**Jang[56]**	2011	Low risk	Low risk	Low risk	Unclear	High risk
**Kono[44]**	2011	Low risk	Low risk	High risk	Unclear	High risk
**Schlapbach[45]**	2011	Low risk	Low risk	Low risk	Unclear	Low risk
**van der Ree[64]**	2011	Low risk	Low risk	Low risk	Unclear	Low risk
**Adams-Chapman[46]**	2013	High risk	Low risk	Low risk	Unclear	Low risk
**De Haan[65]**	2013	Low risk	Low risk	Low risk	Unclear	Low risk
**Dilli[66]**	2013	High risk	Low risk	Low risk	Unclear	Low risk
**Mitha[47]**	2013	Low risk	Low risk	High risk	Unclear	Low risk
**Alshaikh[47]**	2014	Low risk	Low risk	High risk	Low risk	Low risk
**Hentges[58]**	2014	Low risk	Low risk	High risk	Low risk	Low risk
**Yang[59]**	2015	High risk	Low risk	High risk	Unclear	Low risk
**Maruyama[60]**	2016	Low risk	Low risk	High risk	Unclear	Low risk
**Synnes[48]**	2016	High risk	Low risk	High risk	Unclear	High risk
**Young[61]**	2016	Low risk	Low risk	High risk	Unclear	Low risk
**Bright[49]**	2017	Low risk	Low risk	High risk	Low risk	Low risk
**Bolisetty[50]**	2018	Low risk	Low risk	Low risk	Unclear	Low risk
**Zonnenberg[62]**	2019	Low risk	Low risk	Low risk	Low risk	Low risk

Note: assessment was based on the Cochrane's Collaboration tool for assessing risk of bias (See ***Table S2****)*
